# Association between Use of Nutrition Labels and Risk of Chronic Kidney Disease: The Korean National Health and Nutrition Examination Survey (KNHANES) 2008–2019

**DOI:** 10.3390/nu14091731

**Published:** 2022-04-21

**Authors:** Jonghee Kim, Joanne F. Dorgan, Hyesook Kim, Oran Kwon, Yangha Kim, Yuri Kim, Kwang Suk Ko, Yoon Jung Park, Hyesook Park, Seungyoun Jung

**Affiliations:** 1 Department of Clinical Healthcare, Ewha Womans University, Seoul 03760, Korea; jh30522@naver.com; 2 Department of Epidemiology and Public Health, University of Maryland School of Medicine, Baltimore, MD 21201, USA; jdorgan@som.umaryland.edu; 3 Department of Nutritional Science and Food Management, Ewha Womans University, Seoul 03760, Korea; hskim81@ewha.ac.kr (H.K.); orank@ewha.ac.kr (O.K.); yhmoon@ewha.ac.kr (Y.K.); yuri.kim@ewha.ac.kr (Y.K.); kko@ewha.ac.kr (K.S.K.); park.yoonjung@ewha.ac.kr (Y.J.P.); 4 Graduate Program in System Health Science and Engineering, Ewha Womans University, Seoul 03760, Korea; hpark@ewha.ac.kr; 5 Department of Preventive Medicine, College of Medicine, Ewha Womans University, Seoul 07804, Korea

**Keywords:** chronic kidney disease (CKD), CKD prognostic risk, nutrition labels, nutrition label awareness, nutrition label use, Korean National Health and Nutrition Examination Survey

## Abstract

Nutrition labeling on food packages is increasingly found to promote healthier food choices associated with lower risk of chronic kidney disease (CKD). To examine associations between nutrition labels use and CKD risk, we conducted a nationally representative cross-sectional study of 32,080 adults from the 2008–2019 Korean National Health and Nutrition Examination Survey. Nutrition labels use was collected via self-reported questionnaires. Ascertainment and severity of CKD was determined by estimated glomerular filtration rate or proteinuria. In multivariable-adjusted (MV) logistic regression models, increasing awareness and use of nutrition labels was significantly associated with lower CKD risk (MV-adjusted OR “nutrition labels aware and use” group vs. “nutrition labels unaware” group [95% CIs]: 0.75 [0.59–0.95], P_trend_:0.03). This inverse association varied with CKD’s risk of progression, with 21% and 42% reduced risk observed for CKD subtypes with “moderate” and “high” risk of progression, respectively (all P_trend_ ≤ 0.04). Furthermore, the nutrition labels use and CKD risk association significantly differed by age, with 35% reduced risk observed in the older group aged 49 years or older, but not in the younger group (P_interaction_ < 0.001). Our results suggest increasing perception and use of nutrition labels may contribute to CKD prevention and its early asymptomatic progression, especially in older adults.

## 1. Introduction

Chronic kidney disease (CKD) is a long-term condition associated with gradual loss of kidney function [1] and is an increasing public health problem resulting in considerable morbidity and healthcare costs [2,3]. Nearly 700 million people suffer from CKD globally [4], and there was a 29.3% increase in prevalence from 1990 to 2017 [3]. Furthermore, CKD is a clinically silent and asymptomatic disease that is often not detected until later stages, when patients need dialysis or a kidney transplant [5] and are at significantly higher risk of death [6,7]. Thus, primary prevention of CKD, especially by identifying easily modifiable lifestyle factors that inhibit the onset and progression of CKD in the general population poses an effective strategy to reduce its burden [8,9,10].

Nutrition labeling, also known as nutrition (or food) facts labels, is placed on food containers or packaging to provide detailed information about serving size, nutrient content, and the daily percentage values of a food item, including fat, carbohydrate, sugar, sodium, protein, and total energy [11,12]. Since their introduction in the late 1980s to assist in quick, informed, healthier food choices at the time of purchasing food, nutrition labels have rapidly proliferated as a nutritional policy tool in North America, Europe, Asia, and Australasia [13,14,15]. According to the European Food Information Council, a growing number of countries are now mandating nutrition labelling on a much broader range of processed foods and beverage products to promote a healthy food environment [13,14,15]. Indeed, two recent systemic reviews [16,17], a large randomized controlled trial [18], and an international experimental study across 12 countries [19] consistently reported greater perceived healthfulness of food products, healthier food purchasing choices, and greater consumption of better-quality diets high in fruits and vegetables and low in fat, sugar, sodium, and energy among users of nutrition labels, compared with non-users. Such diet consumption has been consistently found to be associated with lower risk of well-known risk factors of CKD [5,20], including obesity [21,22], hypertension [23], and diabetes [24]. Furthermore, recent meta-analyses of 17 prospective cohort studies reported a 30% and 23% lower risk of CKD and albuminuria, the early indicators of kidney damage, among individuals adhering to a healthy diet [25]. Thus, the use of nutrition labels is likely to be associated with lower risk of CKD mediated by its influence on promoting a healthy diet, possibly opening up a novel direction for the development of effective prevention against CKD.

However, to date, only one cross-sectional study of diabetic patients has examined the association between the awareness and use of nutrition labels and renal function, reporting better renal function with greater awareness and use of nutrition labeling [26]. No studies have examined the association of CKD risk with use of nutrition labels among the general population. Therefore, the objective of the present study is to examine our hypothesis that the use of nutrition labels is associated with lower risk of CKD by using data on nutrition labeling awareness and use, demographics, lifestyle, and medical conditions collected by the Korea National Health and Nutrition Survey (KNHANES), a nationally representative cross-sectional survey of Koreans [27]. We have also sought to explore whether the association of nutrition labels use and risk of CKD varies according to CKD subtypes defined by its risk of progression and population characteristics as a secondary analysis.

## 2. Methods

### 2.1. Study Design and Population

This study was conducted using data from the KNHANES from 2008 to 2019 [27]. In brief, the KNHANES is a continuous cross-sectional survey of non-institutionalized South Koreans of all ages that has been conducted by the Korea Centers for Disease Control (KCDC) since 1998. The KNHANES uses a multi-stage clustered probability sampling design to obtain data on demographic and socioeconomic status, health-related lifestyles, and medical conditions from a nationally representative population and monitors trends in health risk factors and the prevalence of major chronic diseases in South Korea [27]. The KNHANES combines health interviews and nutrition surveys with physical examinations and laboratory tests of biochemical markers. Written informed consent is obtained from all participants prior to their enrollment in the survey. The institutional review boards of KCDC have approved all KNHANES protocols. The present study was exempt from review by the institutional review board of Ewha Womans University because it uses de-identified and publicly available data (IRB no. 202112-0001-01).

**Figure 1:**
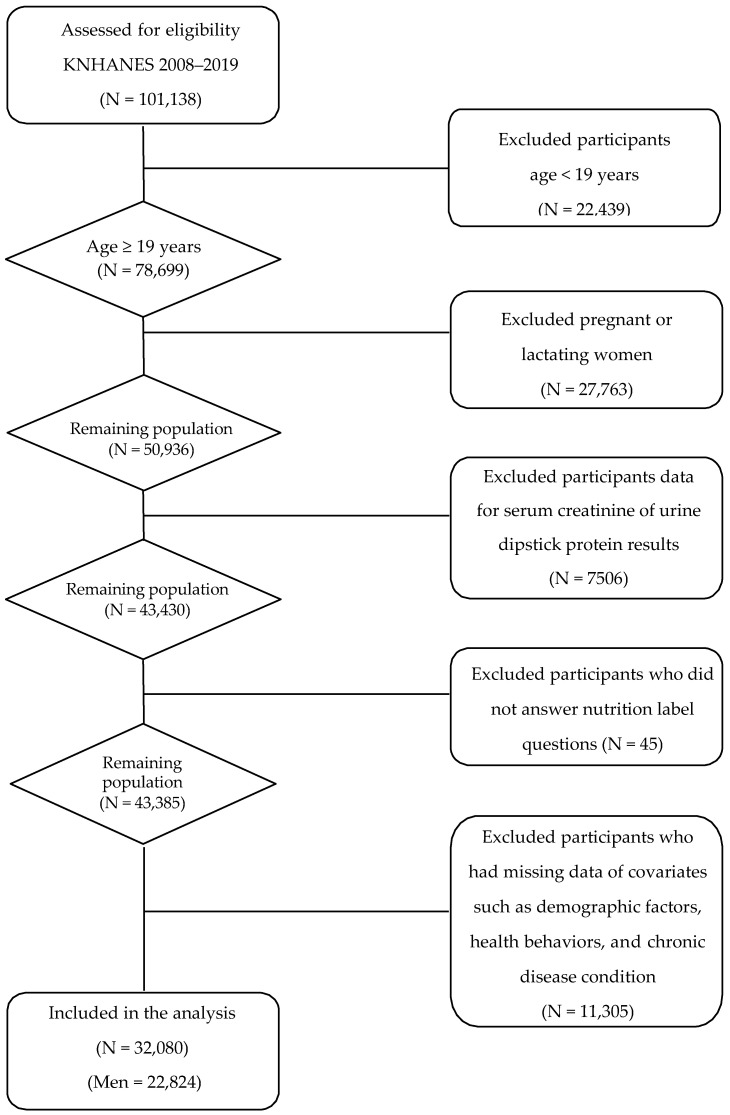
A flow diagram of the study subjects.

For the present analyses, we pooled the annual KNHANES data collected cross-sectionally between 2008 and 2009. Of 101,138 individuals who participated in the KNHANES between 2008 and 2019, we excluded those who: (1) were under 19 years of age (N = 22,439), (2) were pregnant or lactating (N = 27,763), (3) had no serum creatinine measurements or urine dipstick protein results necessary for CKD ascertainment (N = 7506), (4) did not answer nutrition label questions (N = 45), (5) had implausible energy intake (<500 kcal/d or >5000 kcal/d; N = 8021), and (6) had missing data on covariates included in a primary multivariable model (N = 3284) (Figure 1). Consequently, the final sample size for the present analysis was 32,080 adults.

### 2.2. Assessment of Use of Nutrition Labels

Data on the use of nutrition labels was collected via self-reported questionnaires. The questionnaire specifically asked: “Are you aware of the nutrition labels?” Those who answered “Yes” to the question were asked, as follow-up: “Do you read nutrition labels when you purchase food items?” Based on the responses to the questions, we classified the participants into three groups, each with increasing awareness and use of nutrition labels. Those who answered “No” to the first question, on nutrition labeling awareness, were classified into the “Nutrition labels unaware” group (the lowest level of nutrition labels use). Those who answered “yes” to the first question but “No” to the follow-up question on nutrition labels use at the time of purchasing a food product were classified into the “Nutrition labels aware only” group. Those who answered “yes” to both questions were classified into the “Nutrition labels aware and use” group (the highest level of nutrition labels use). 

### 2.3. Ascertainment of CKD

CKD status was defined using measures of kidney function or kidney damage as recommended by “Kidney Disease: Improving Global Outcomes (KDIGO)” [5]. Glomerular filtration rate is the flow rate of filtered fluid through the kidney and serves as the best indicator of overall kidney function. We estimated GFR (eGFR), using the Chronic Kidney Disease Epidemiology Collaboration (CKD-EPI) equation [28,29,30], which incorporates sex, age, race, and serum level of creatinine. The eGFR values were classified into six eGFR categories as follows: G1: ≥90 mL/min/1.73 m^2^; G2: 60–89 mL/min/1.73 m^2^; G3a: 45–59 mL/min/1.73 m^2^; G3b: 30–44 mL/min/1.73 m^2^; G4; 15–29 mL/min/1.73 m^2^; and G5; <15 mL/min/1.73 m^2^ [5]. Proteinuria serves as a surrogate marker for kidney damage and kidney disease progression [31]. The presence and levels of proteinuria were determined based on urine dipstick results. The values of “negative to trace” protein results were defined as “normal to mild increase” proteinuria (A1); “trace to +” as “moderate increase” proteinuria (A2); and “+ or greater” as “severe increase” proteinuria (A3) [5,32]. 

Then, following KDIGO guidelines [5], CKD cases were confirmed if participants belonged to G3a, G3b, G4, or G5 eGFR groups (eGFR < 60 mL/min per 1.73 m^2^) or A2 or A3 proteinuria groups (as “moderate increase” or “severe increase” proteinuria) [5]. CKD cases were further classified into three groups, each reflecting their prognostic risk for progression, morbidity, and mortality based on eGFR and proteinuria levels [5]. These were “moderately increased risk” (G3a and A1; G1–G2 and A2), “high risk” (G3b and A1; G3a and A2; or G1–G2 and A3), and “very high risk” (G4–G5; G3b and A2–A3; or G3a and A3). CKD cases were also grouped following the conventional staging definition, namely stage I (G1 and A2 or A3), stage II (G2 and A2 or A3), stage III (G3), stage IV (G4), and stage V (G5). CKD cases with stages I, II, and III were defined as non-advanced CKD, whereas CKD cases with stages IV and V were defined as advanced CKD [33,34,35,36]. As a result, we ascertained a total of 1437 CKD cases, of which 1015 were “moderately increased risk”, 299 were “high risk”, and 123 were “very high risk” cases, while1376 were non-advanced and 61 were advanced CKD cases. 

### 2.4. Assessment of Covariates

Information on participants’ sociodemographics (sex, age, household income, region, education), health-related lifestyle (smoking status and drinking behavior), and medical conditions was collected via standardized self-reported questionnaires. Physical activity was measured using the international physical activity questionnaire—short form [37,38]. Participants were defined as being active if engaging in ≥150 min moderate physical activity or ≥75 min vigorous physical activity per week; otherwise, they were defined as being inactive. Body weight and height were measured by a trained examiner, and body mass index (BMI) was calculated as weight (kg) divided by the square of the height (m^2^). Dietary data were collected via 24 h recalls assisted by trained interviewers, and daily nutrients intake from food consumed was calculated based on the national standard food composition table [39]. Blood pressure level was measured with a standard mercury sphygmomanometer. Hypertension was defined as: (1) ≥130 mm Hg systolic or ≥80 mm Hg diastolic blood pressure from an average of two blood pressure readings [40]; or (2) use of antihypertensive medication. Diabetes was defined as: (1) fasting plasma glucose level ≥126 mg/dl or hemoglobin A_1C_ ≥ 6.5%; or (2) use of oral hypoglycemic agents. Hyperglycemia was determined if fasting plasma glucose ≥ 126 mg/dL. Hypercholesterolemia was defined as: (1) fasting total serum cholesterol level ≥240 mg/mL; or (2) use of lipid-lowering medications. 

### 2.5. Statistical Analyses

To account for the multistage sampling design of KNHANES and produce unbiased national estimates, the sampling weights were applied in all analyses. Study population characteristics according to the use of nutrition labels were summarized as mean ± standard deviation (SD) by using the SAS SURVEYMEANS procedure for continuous variables and numbers and percentages by using the SAS SURVEYFREQ procedure for categorical variables.

Logistic regression models were used to calculate odds ratios (OR) and 95% confidence intervals (95% CI) to evaluate the association between use of nutrition labels and risk of overall CKD and CKD subtypes, defined by progression risk, using the SAS SURVERYLOGISTIC procedure. The multivariable model was adjusted for potential confounding factors of well-known risk factors for CKD [6,20,41] as follows: age (years, continuous), sex (men, women), household income (low, middle-low, middle-high, high), region (urban, rural), educational level (less than elementary school, middle school, high school, college or higher), smoking status (not a current smoker, current smoker), high-risk drinking (not a binge drinker, binge drinker), physical activity (inactive, active), obesity status (BMI < 25.0 kg/m^2^, BMI ≥ 25.0 kg/m^2^) [42], hypertension (no hypertension, hypertension), diabetes (no diabetes, diabetes), hypercholesterolemia (no hypercholesterolemia, hypercholesterolemia), hyperglycemia (no hyperglycemia, hyperglycemia), and intake of total energy (kcal/d, continuous). Our primary model did not adjust for fruit and vegetable consumption, since this may be on the causal pathway between use of nutrition labels and risk of CKD. However, we tested the robustness of results to additionally include fruit and vegetable consumption in sensitivity analyses. Other sensitivity analyses conducted were including additional comorbid conditions in the model and excluding cases with end-stage renal condition, defined as stage V. A test for trend was conducted by including the ordinal variable of nutrition labels use as a continuous term and evaluating its statistical significance using a Wald test. To examine whether associations varied by population characteristics, we also conducted analyses stratified by sex [43,44,45,46,47,48], age [26,49], obesity status [50,51,52], hypertension [52,53], and diabetes [52,54]. The statistical significance of the heterogeneity of associations was evaluated using the Wald test for the cross-product term between use of nutrition labels and stratification factors.

Analyses were performed using SAS version 9.4 (SAS Institute, Inc., Cary, NC, USA). All statistical tests were two-sided at a significance level <0.05. 

## 3. Results

Table 1 presents the characteristics of the study population. Mean age and total energy intake of the study population was 43.2 ± 0.1 years and 2191.2 ± 6.7 kcal/d. The majority of the study population were men (77.2%), had at least middle-high income level (62.7%), lived in an urban area (83.9%), were married (62.3%), and were not currently smoking (63.9%) or binge drinking (83.3%), although many were physically inactive (62.1%). In addition, the majority were not overweight (65.3%) and did not have hypertension (59.5%) or diabetes (91.0%). With respect to the level of awareness and use of nutrition labels, 8835 (20.3%) were in the “Unaware group”, 16,716 (56.3%) were in the “Aware only” group, and 6529 (23.4%) were in the “Aware and use” group. Participants in the “Aware and use” group were more likely to be younger and female and had greater income and higher education, and lived in urban areas, compared to those in the “Unaware group”. In terms of lifestyle and medical conditions, those in the “Aware and use” group were less likely to smoke and binge drink, were more physically active, and had less hypertension or diabetes, compared to those in the “Unaware group”.

Associations between use of nutrition labels and risk of CKD were similar in age-adjusted and multivariable-adjusted models, and suggested significantly lower risk of CKD with increasing awareness and use of nutrition labels (all P_-trend_ ≤ 0.03) (Table 2). The multivariable-adjusted OR (95% CI) were 0.80 (0.70–0.93) for the “Aware only” group and 0.75 (0.59–0.95) for the “Aware and use” group compared to “Unaware” group. Further adjustment for fruit and vegetable consumption or comorbid conditions did not alter the results materially (data not shown). Restricting analyses to those without end-stage renal disease also yielded similar results (data not shown). 

CKD progression varies according to levels of eGFR and albuminuria [5]. We, therefore, examined whether the observed lower risk of CKD with the use of nutrition labels differed by the severity of CKD. Table 3 presents the results of the analyses with CKD cases classified by the potential for CKD progression using the new composite ranking system of KDIGO [5]. There was a suggestion of a stronger inverse association with CKD cases with moderate or high risk of progression (all P-trend ≤ 0.04) compared to CKD cases with very high risk (Table 3). The multivariable-adjusted OR (95% CI) comparing the “Aware and use” group to the “Unaware” group was 0.79 (0.59–1.05) for CKD cases with moderate risk of progression and 0.58 (0.35–0.96) for CKD cases with high risk of progression, whereas there was no association with CKD cases with very high risk of progression. Results from additional analyses examining the associations with CKD subgroups defined by the conventional staging system, which uses eGFR levels alone [33,34,35,36], were also similar, showing the apparent inverse association of nutrition label use with CKD cases with lower stages than those with higher stages (Supplementary Material Table S1).

In analyses stratified by population characteristics (Table 4), obesity, hypertension, and diabetes status did not significantly modify the association between use of nutrition labels and risk of CKD. Sex also did not significantly modify the association; the results did not change materially when we further explored effect modification by sex across age groups defined by using the age 55 years cut-off (Supplementary Material Table S2) [55]. However, age significantly modified the association between nutrition label use and risk of CKD (P-interaction: <0.001). Specifically, in the group of 49 years old or older, the multivariable OR (95% CIs) was 0.80 (0.67–0.94) for the “Aware only” group and 0.65 (0.46–0.92) for the “Aware and use” group, compared to the “Unaware” group (P-trend: 0.002), whereas no significant associations were observed in younger adults.

## 4. Discussion

In this cross-sectional study of nationally representative data in Korea, we observed a significantly lower risk of CKD with increasing awareness and use of nutrition labels. This inverse association was found to be more pronounced with CKD cases with moderate and high risk of progression, compared with CKD cases with very high risk of progression. Furthermore, the association between the use of nutrition labels and risk of CKD varied by age with a strong, significant inverse association observed in the older group, but not in the younger group. None of the other factors examined, including sex and obesity, hypertension, and diabetes status, significantly modified the association between use of nutrition labels and CKD risk. To the best of our knowledge, the present study is the first comprehensive, large epidemiologic analysis to examine the association between use of nutrition labels and risk of CKD overall and CKD subtypes defined by potential for progression and by subgroup characteristics. Our results suggest that diet modification, possibly through use of nutrition labels, may contribute to the prevention of CKD overall and its early asymptomatic progression, especially in older individuals. 

To date, there has been sparse evidence for the association between nutrition labels use and the risk of CKD. Since the inception of nutrition labeling in the late 1980s, the majority of previous studies examining nutrition labels have primarily focused on demonstrating their impact on healthier dietary behavior [56] and improvements of diet quality [57,58,59,60]. Relatively few epidemiologic studies have examined the disease risk associated with the use of nutrition labels [26,51,60,61,62,63,64,65,66], and most examined associations with metabolic conditions [60,63,64,65,66,67,68] and diabetes [51,62,65]. To our knowledge, only one cross-sectional study of diabetic patients reported an association between the use of nutrition labels and renal function decline [26]. Consistent with our results, the study reported that unawareness of nutrition labeling is associated with significantly greater loss of renal function [26]. 

In addition, increasing evidence strongly indicates that diet affects kidney function and CKD development via its effects on oxidative stress, inflammation, lipid and glucose metabolism, and known comorbid risk factors of CKD such as hypertension and diabetes [25,69,70]. Promotion of a healthy diet by nutrition labelling [16] supports the plausibility of the inverse association between nutrition labels use and risk of CKD that we observed. Nutrition labeling in Korea, as in other countries, displays the contents, serving size, and percent of daily values of nutrient profiles to limit or encourage consumption, recommended by the WHO, for sodium, saturated fat, trans-fatty acids, sugar, cholesterol, and protein on the packaging of food items. Substantial evidence indicates that being informed of a food’s nutritional quality via the use of nutrition labels may direct food purchase intentions towards healthier food choices [16,17,71]. Indeed, in large national studies in Korea [58] and the USA [59,60,72], nutrition label users were found to consume lower amounts of total energy, total fat, saturated fat, cholesterol, sodium, and sugars and greater amounts of fiber and more fruits, vegetables, and whole grains, and fewer sugar-sweetened beverages, all of which characterize a healthy diet quality, compared with non-users. Greater adherence to such a healthy diet [21,22,23,24] and use of nutrition labels [51,61,62,63,64,65,67,68] have been associated with lower risk of CKD-related clinical factors, including obesity [21,22,67], metabolic syndrome [63,68], dyslipidemia [64], diabetes [24,51,61,62], and hypertension [23,65]. Furthermore, a recent large meta-analysis of 18 prospective cohort studies involving 630,108 adults reported that a healthy dietary pattern is associated with 30% and 23% lower risk of CKD and albuminuria, a marker of kidney damage [25], respectively. Our current findings on CKD extend the increasing population-based evidence supporting the health benefits of nutrition labeling for prevention and management of CKD in addition to diet-related chronic diseases [26,51,60,61,62,63,64,65,66].

CKD is asymptomatic and progresses silently to advanced stages, often being detected too late at fatal stages [5]. Identification of risk factors associated with CKD cases with varied potential of progression may thus open a novel venue for the primary prevention of CKD as well as the development of strategic planning to delay its progression, but it has been an understudied area. To date, no prior studies have examined the association between use of nutrition labeling and risk of CKD progression, as our study has, but there are several lines of evidence that the use of nutrition labeling may preserve existing renal function and delay CKD progression. For example, a cross-sectional study previously reported a dose-response trend with use of nutrition labels and eGFR levels, a strong indicator of CKD progression [26]. Furthermore, several prospective studies of healthy diet, possibly being promoted by nutrition labeling, consistently reported attenuated eGFR decline over study follow-up [73,74,75] or lower risk of CKD progression to end-stage renal disease or mortality [73]. 

Similarly, we observed a downward trend in the association, with 21% and 41% reduced risk observed with CKD subtypes with increasing potential for “moderate” to “high” risk progression, respectively, among the “Aware and use group” compared with the “Unaware group”. However, the association was not observed with very high risk of progression, which was unexpected. One possible explanation for this is that individuals with CKD subtypes with very high risk of progression are likely to suffer from other complications, such as acute kidney injuries or kidney stones, undergoing rapid deterioration of kidney function [76] and possibly being at stages too late for having health benefits derived from diet. Alternatively, individuals tend to change their diets consciously, regardless of use of nutrition labels, as CKD progresses to end stage with increasing symptoms and clinical diagnosis, attenuating the association. Despite these possibilities, we had a relatively small number of CKD cases with very high risk for progression limiting power and the results could be due to chance. A larger prospective study with a long period of follow-up is necessary to better elucidate the association between nutrition labels use and the risk of CKD subtypes with varying potential to progress. 

Interestingly, we observed a stronger inverse association between nutrition labels use and the risk of CKD in the older group than in the younger group. The mechanisms of such greater possible benefit of nutrition labels use observed in the older population remain unclear. Nonetheless, age-based disparities in CKD risk, although limited, were previously reported, possibly suggesting biologic heterogeneity of CKD cases by age [26,49]. For example, our results are consistent with the study of diabetic patients that found less loss of renal function associated with nutritional labels use among older rather than younger diabetic populations [26]. Similarly, water intake [49] was more strongly associated with the risk of renal impairment in the older group than in the younger group. Several possibilities may explain these stronger associations of diet-related factors with CKD risk in the older group. With aging, the kidney is known to experience progressive structural changes and functional declines, characterized as kidney cyst formation, renal volume decrease, nephrosclerosis, glomerular basement membrane thickening, and nephron loss [77]. It is possible that the older population, compared to the younger population, is more susceptible to dietary insults, suggestively explaining the greater benefit of healthy diet, and thus the use of nutrition labels with aging. In addition, considerable evidence reported heterogeneity in the etiology, risk factors, and progression of CKD by age [78,79,80]. Notably, CKD occurring at younger ages is more likely due to congenital anomalies of the kidney and urinary tract rather than diet-related CKD-related clinical factors, including diabetic nephropathy or hypertension. Our observation may reflect greater hereditary influence on the development of CKD at younger ages than other lifestyle factors, including diet. Alternatively, our result might also arise from more active and frequent uses of nutrition labels while purchasing foods among the older rather than younger population, but we did not have such data to explore this hypothesis. Finally, our results could also be due to chance given the limited number of young CKD cases resulting in low power. Large cohort studies with more detailed information on nutrition labels use across various domains, such as frequency, duration, and impact on decision-making at the time of food purchasing, are needed to further explore differences in associations by age. 

Our study had several limitations that warrant discussion. First, the cross-sectional nature of the study design limits drawing causal inferences from the association between nutrition labels use and the risk of CKD that we observed. Second, the self-reported data of awareness and use of nutrition labels in the KHANES do not provide information on frequency, as in other studies [60,81], or the specific use of information on nutrition labels, such as use of nutrition facts panel, serving size, and percent daily values, thereby limiting the identification of the most important elements on nutrition labels associated with CKD risk. Third, we cannot rule out that use of nutrition labels serves as a proxy for health-conscious behavior. Nonetheless, our model fully adjusted for known health-related lifestyle factors. Additional adjustment for fruit and vegetable consumption also did not materially change our results. Fourth, the clinical definition of CKD is kidney damage or abnormalities of kidney function present for >3 months. With a single measurement of creatinine, we were not able to measure chronicity of CKD, possibly resulting in an overestimation of CKD cases. However, the KNHANES is a study of a community-dwelling population whose proportion with acute kidney injury is likely to be low [82]. We assessed kidney damage by determining proteinuria, alternative to albuminuria—a gold standard measure for kidney damage—using dipstick results. Nonetheless, KDIGO has long supported the use of proteinuria results as a substitute for albuminuria [5,41]. Furthermore, aligning with the KDIGO guideline, the results of recent international collaborative meta-analyses of 919,833 individuals from 33 cohorts also support the consistency between protein dipstick categories and albumin–creatinine ratio categories [32]. Finally, we cannot rule out residual and unmeasured confounding, including family history of CKD. Nonetheless, given the approximately 13% prevalence of CKD in South Korea, our results from KNHANES, a nationally representative sample, are likely to be driven mostly by participants without a family history of CKD and the confounding by such a factor is expected to be low.

Our study also had several strengths. It is the first to examine the association between use and awareness of nutrition labels and risk of CKD in a large, nationally representative sample of the general population in South Korea. Furthermore, our analysis is distinct from other prior studies of CKD as it applied the most recent, novel classification of CKD defined by KIDO, namely “moderate increased risk,” “high risk,” and “very high risk,” that best represents its progression potential to adverse outcome [83]. With this approach, we were the first to estimate association of nutrition labels use across multiple levels of CKD progression potential. Taking advantage of the rich, high-quality data on lifestyle, anthropometrics, and medical histories collected from standardized protocols in the KNHANES, we were also able to comprehensively adjust for possible confounding factors known to be associated with CKD and conduct detailed analyses of the association overall and by subtypes of CKD, as well as by population characteristics. Through several sensitivity analyses, we demonstrated the robustness of our results. Finally, our findings are of high public health significance, being generalizable to Korean adults. They also add to the increasing scientific evidence of the health benefit of nutrition labeling, and underscore the great potential of nutrition labeling policy.

## 5. Conclusions

In this first nationally representative cross-sectional study of the general population in South Korea, the use of nutrition labels was found to be associated with lower risk of CKD with stronger associations observed in CKD cases with moderate and high risk of progression and among older adults. With increasing evidence that strongly supports the favorable impact of nutrition labeling on behavioral change towards healthier food purchasing [84], nutrition labels currently appear on the majority of food packages and are expected to be further extended as a global key regulatory nutritional policy. At this junction of nutrition labeling expansion, our results further provide novel insights on nutrition labeling as a simple, effective tool in the prevention and management of CKD. Further prospective cohort studies with detailed uses of the information on nutrition labels are warranted to confirm our observed associations between the use of labeling and CKD risk. 

## Figures and Tables

**Table 1:** Characteristics ^#^ of study participants (N = 32,080).

Characteristics	Total	Use of Nutrition Labels		
		Unaware	Aware Only	Aware and Use
	(N = 32,080)	(N = 8835)	(N = 16,716)	(N = 6529)
**Demographic factors**				
Age, years	43.2 ± 0.1	57.3 ± 0.3	41.4 ± 0.2	35.3 ± 0.2
Sex				
Men	22,824 (77.2%)	7092 (87.5%)	12,352 (80.1%)	3380 (61.4%)
Women	9256 (22.8%)	1743 (12.5%)	4364 (19.9%)	3149 (38.6%)
Household income				
Low	5799 (13.2%)	3180 (27.4%)	2030 (9.7%)	589 (9.2%)
Middle-Low	7923 (24.2%)	2418 (27.4%)	4059 (23.7%)	1446 (22.5%)
Middle-High	8889 (30.1%)	1801 (24.6%)	5112 (32.1%)	1976 (30.3%)
High	9469 (32.6%)	1436 (20.7%)	5515 (34.6%)	2518 (38.0%)
Region				
Urban	25,005 (83.9%)	5778 (73.9%)	13,557 (85.4%)	5670 (89.0%)
Rural	7075 (16.1%)	3057 (26.1%)	3159 (14.6%)	859 (11.0%)
Educational level				
Less than elementary school	6224 (11.3%)	4173 (34.8%)	1870 (6.7%)	181 (1.9%)
Middle school	3353 (8.2%)	1392 (15.6%)	1626 (7.5%)	335 (3.4%)
High school	11,212 (39.6%)	2062 (30.3%)	6513 (41.9%)	2637 (42.1%)
College or higher	11,291 (40.9%)	1208 (19.3%)	6707 (43.9%)	3376 (52.6%)
Marital status				
Single	7173 (32.2%)	600 (12.6%)	3955 (32.1%)	2618 (49.4%)
Married	22,293 (62.3%)	6873 (75.7%)	11,753 (63.5%)	3667 (47.8%)
Divorced/Widow/Widower	2614 (5.5%)	1362 (11.7%)	1008 (4.4%)	244 (2.8%)
**Dietary intake**				
Total energy intake, kcal/d	2191.2 ± 6.7	2112.9 ± 13.4	2249.1 ± 8.7	2119.8 ± 13.2
**Healthy behavior factors**				
Smoking				
No current smoker	21,505 (63.9%)	5879 (62.0%)	10,614 (60.3%)	5012 (74.2%)
Current smoker	10,575 (36.1%)	2956 (38.0%)	6102 (39.7%)	1517 (25.8%)
Drinking				
Non-binge drinker	27,446 (83.3%)	7703 (83.4%)	13,950 (81.6%)	5793 (87.3%)
Binge drinker	4634 (16.7%)	1132 (16.6%)	2766 (18.4%)	736 (12.7%)
Physical activity				
Inactive	21,067 (62.1%)	6363 (69.4%)	10,892 (62.9%)	3812 (54.1%)
Active	11,013 (37.9%)	2472 (30.6%)	5824 (37.1%)	2717 (45.9%)
**Chronic disease factors**				
Obesity				
BMI < 25.0 kg/m^2^	21,174 (65.3%)	5859 (65.4%)	10,879 (64.5%)	4436 (67.1%)
BMI ≥ 25.0 kg/m^2^	10,906 (34.7%)	2976 (34.6%)	5839 (35.5%)	2093 (32.9%)
Hypertension				
No hypertension	15,306 (51.5%)	2893 (36.0%)	8252 (51.9%)	4161 (64.1%)
Hypertension	16,774 (48.5%)	5942 (64.0%)	8464 (48.1%)	2368 (35.9%)
Diabetes				
No diabetes	28,336 (91.0%)	7310 (83.4%)	15,113 (92.4%)	6116 (94.6%)
Diabetes	3744 (9.0%)	1525 (16.6%)	1603 (7.6%)	413 (5.4%)
Hypercholesterolemia				
No hypercholesterolemia	27,584 (87.4%)	7310 (83.4%)	14,442 (87.6%)	5832 (90.4%)
Hypercholesterolemia	4496 (12.6%)	1525 (16.6%)	2274 (12.4%)	697 (9.6%)
Hyperglycemia				
No hyperglycemia	29,587 (93.6%)	7762 (88.7%)	15,588 (94.4%)	6237 (96.0%)
Hyperglycemia	2493 (6.4%)	1073 (11.3%)	1128 (5.6%)	292 (4.0%)

Abbreviation: BMI, body mass index. ^#^ Values are mean ± se or N (percentages).

**Table 2:** Multivariable-adjusted ^$^ odds ratio (95% confidence intervals) of overall CKD by use of nutrition labels.

Use of Nutrition Labels				
	Unaware Group *	Aware only Group **	Aware and Use Group ***	P for Trend ****
Cases/Non-cases	755/8080	551/16,165	131/6398	
Age-adjusted	1.00 (ref)	0.81 (0.70–0.93)	0.78 (0.62–0.99)	0.009
Multivariable-adjusted	1.00 (ref)	0.80 (0.70–0.93)	0.75 (0.59–0.95)	0.03

Abbreviation: CKD, chronic kidney disease. ^$^ Adjusted for age (years, continuous), sex (men, women), household income (low, middle-low, middle-high, high), region (urban, rural), educational level (less than elementary school, middle school, high school, college or higher), smoking (no current, current smoker), high-risk drinking (no-binge drinker, binge drinker), physical activity (inactive, active), obesity (BMI < 25.0 kg/m^2^, BMI ≥ 25.0 kg/m^2^), hypertension (no hypertension, hypertension), diabetes (no diabetes, diabetes), hypercholesterolemia (no hypercholesterolemia, hypercholesterolemia), hyperglycemia (no hyperglycemia, hyperglycemia), and total energy intake (kcal/d, continuous). * A group was not aware of nutrition labels. ** A group was aware of nutrition labels but did not use them when purchasing food items. *** A group was aware of nutrition labels and used them when purchasing food items. **** P for trend was tested from model including the ordinal variable of nutrition labels use as a continuous term and using Wald test for it.

**Table 3:** Multivariable-adjusted ^$^ odds ratio (95% confidence intervals) of CKD subtypes defined by its risk of progression by use of nutrition labels.

Use of Nutrition Labels				
	Unaware Group *	Aware only Group **	Aware and Use Group ***	P for Trend ****
**CKD cases with moderate risk of progression ^†^**				
Cases/Non-cases	530/8080	392/16,165	93/6398	
Age-adjusted	1.00 (ref)	0.81 (0.68–0.96)	0.81 (0.62–1.07)	0.05
Multivariable-adjusted	1.00 (ref)	0.82 (0.69–0.97)	0.79 (0.59–1.05)	0.04
**CKD cases with high risk of progression ^††^**				
Cases/Non-cases	156/8080	117/16,165	26/6398	
Age-adjusted	1.00 (ref)	0.79 (0.61–1.03)	0.64 (0.39–1.06)	0.03
Multivariable-adjusted	1.00 (ref)	0.75 (0.57–0.99)	0.58 (0.35–0.96)	0.01
**CKD cases with very high risk of progression ^†††^**				
Cases/Non-cases	69/8080	42/16,165	12/6398	
Age-adjusted	1.00 (ref)	0.84 (0.52–1.38)	0.92 (0.42–2.01)	0.68
Multivariable-adjusted	1.00 (ref)	0.81 (0.52–1.27)	0.91 (0.43–1.93)	0.61

Abbreviation: CKD, chronic kidney disease. ^$^ Adjusted for the same variables included in the multivariable model of Table 2. * A group was not aware of nutrition labels. ** A group was aware of nutrition labels but did not use them when purchasing food items. *** A group was aware of nutrition labels and used them when purchasing food items. **** P for trend was tested from model including the ordinal variable of nutrition labels use as a continuous term and using the Wald test for it. **^†^** CKD cases with moderate risk of progression were defined if participants belonged to any of the following groups: (i) eGFR within 45–59 mL/min/1.73 m^2^ and “normal to mild increase” proteinuria; or (ii) eGFR ≥ 60 mL/min/1.73 m^2^ and “moderate increase” proteinuria. **^††^** CKD cases with high risk of progression were defined if participants belonged to any of the following groups: (i) eGFR within 30–44 mL/min/1.73 m^2^ and “normal to mild increase” proteinuria; (ii) eGFR within 45–59 mL/min/1.73 m^2^ and “moderate increase” proteinuria; or (iii) eGFR ≥60 mL/min/1.73 m^2^ and “severe increase” proteinuria. **^†††^** CKD cases with very high risk of progression were defined if participants belonged to any of the following groups: (i) eGFR <30 mL/min/1.73 m^2^; (ii) eGFR within 30–44 mL/min/1.73 m^2^ and moderate or severe increase” proteinuria; or (iii) eGFR within 45–59 mL/min/1.73 m^2^ and “severe increase” proteinuria.

**Table 4:** Multivariable-adjusted ^$^ odds ratio (95% confidence intervals) of overall CKD by use of nutrition labels according to population characteristics.

Stratification Factors	Cases /Non-Cases	Use of Nutrition Labels			P for Trend ****	P for Interaction *****
		Unaware Group *	Aware only Group **	Aware and Use Group ***		
**Sex**						
Men	1173/21,651	1.00 (ref)	0.85 (0.73–1.00)	0.87 (0.66–1.13)	0.12	0.60
Women	264/8992	1.00 (ref)	0.57 (0.38–0.86)	0.35 (0.20–0.63)	<0.001	
**Age**						
<49 years	242/15,644	1.00 (ref)	2.06 (1.08–3.92)	1.66 (0.82–3.34)	0.79	<0.001
≥49 years	1195/14,999	1.00 (ref)	0.80 (0.67–0.94)	0.65 (0.46–0.92)	0.002	
**Obesity**						
BMI < 25.0 kg/m^2^	839/20,338	1.00 (ref)	0.70 (0.57–0.85)	0.67 (0.48–0.93)	0.003	0.50
BMI ≥ 25.0 kg/m^2^	601/10,305	1.00 (ref)	0.98 (0.79–1.22)	0.88 (0.61–1.27)	0.50	
Hypertension ******						
Normal	238/13,255	1.00 (ref)	0.61 (0.41–0.90)	0.65 (0.39–1.07)	0.17	0.26
Elevated	63/1750	1.00 (ref)	0.74 (0.40–1.35)	0.22 (0.041–1.15)	0.03	
Hypertension stage 1	400/8304	1.00 (ref)	0.97 (0.74–1.28)	0.77 (0.49–1.23)	0.29	
≥Hypertension stage 2	445/5353	1.00 (ref)	0.97 (0.76–1.25)	0.85 (0.54–1.34)	0.49	
**Diabetes**						
No diabetes	884/27,452	1.00 (ref)	0.76 (0.64–0.92)	0.68 (0.51–0.91)	0.003	0.37
Diabetes	553/3191	1.00 (ref)	0.93 (0.73–1.18)	0.90 (0.58–1.39)	0.47	

Abbreviation: CKD, chronic kidney disease. ^$^ Adjusted for the same variables included in the multivariable model of Table 2. * A group was not aware of nutrition labels. ** A group was aware of nutrition labels but did not use them when purchasing food items. *** A group was aware of nutrition labels and used them when purchasing food items. **** P for trend was tested from model including the ordinal variable of nutrition labels use as a continuous term and using the Wald test for it. ***** P for interaction was tested using the cross-product term between use of nutrition labels and stratification factors. ****** Individuals without hypertension were categorized into normal and elevated groups using the following definition: systolic blood pressure < 120 mmHg and diastolic blood pressure < 80 mmHg for normal group, and systolic blood pressure ≥120–<130 mmHg and diastolic blood pressure < 80 mmHg for elevated group. Individuals with hypertension were categorized into hypertension stage 1 and ≥ hypertension stage 2 groups using the following definition: systolic blood pressure ≥130–<140 mmHg or diastolic blood pressure ≥80–<90 mmHg for hypertension stage 1 group, and systolic blood pressure ≥ 140 mmHg or diastolic blood pressure ≥ 90 mmHg for ≥ hypertension stage 2 group.

## Data Availability

The data from the KNHANES are available to the public and can be obtained after request from the website https://knhanes.kdca.go.kr/knhanes/main.do, accessed on 14 April 2022.

## Supplementary Materials

The following supporting information can be downloaded at: https://www.mdpi.com/article/10.3390/nu14091731/s1, Table S1: Multivariable-adjusted^$^ odds ratio (95% confidence intervals) of CKD defined by stages by use of nutrition labels, Table S2: Multivariable-adjusted^$^ odds ratio (95% confidence intervals) of overall CKD by use of nutrition labels according to sex and age.

## Abbreviations

BMI: body mass index; CKD, chronic kidney disease; CKD-EPI, Chronic Kidney Disease Epidemiology Collaboration; eGFR, estimated glomerular filtration rate; KDIGO, Kidney Disease: Improving Global Outcomes; KNHANES, the Korea National Health and Nutrition Survey.
