# Supplementary material for: Association between Use of Nutrition Labels and Risk of Chronic Kidney Disease: The Korean National Health and Nutrition Examination Survey (KNHANES) 2008–2019
Source: Nutrients. 2022 Apr 21;14(9):1731. doi: 10.3390/nu14091731 (PMC9105550; doi:10.3390/nu14091731)
Supplement: Supplementary file 1 [file nutrients-14-01731-s001.zip › nutrients-1641100-supplementary.pdf]

Supplementary Material Table S1. Multivariable-adjusted$ odds ratio (95% confidence intervals) of CKD subtypes defined by stages by use of nutrition labels

	Use of nutrition labels			
	Unaware group*	Aware only group**	Aware and use group***	P for trend****
**Non-advanced CKD‡**				
Cases / Non-cases	722 / 8,8080	528 / 16,165	126 / 6,398	
Age-adjusted	1.00 (ref)	0.81 (0.70-0.94)	0.80 (0.63-1.01)	0.02
Multivariable-adjusted	1.00 (ref)	0.81 (0.70-0.93)	0.76 (0.59-0.98)	0.009
**Advanced CKD‡‡**				
Cases / Non-cases	33 / 8,080	23 / 16,165	5 / 6,398	
Age-adjusted	1.00 (ref)	0.71 (0.36-1.38)	0.49 (0.14-1.69)	0.19
Multivariable-adjusted	1.00 (ref)	0.72 (0.38-1.36)	0.47 (0.14-1.60)	0.15

Abbreviation: CKD, chronic kidney disease.

$ Adjusted for the same variables included in the multivariable model of table 2.

* A group was not aware of nutrition labels.
** A group was aware of nutrition labels but didn’t use to them.
*** A group was aware of nutrition labels and used to them.

**** P for trend was tested from model including the ordinal variable of nutrition labels use as a continuous term and using the Wald test of it.

‡ Non-advanced CKD was defined for CKD with stages I, II, and III.

‡‡ Advanced CKD was defined for CKD with stages IV and V.

Supplementary Material Table S2. Multivariable-adjusted$ odds ratio (95% confidence intervals) of overall CKD by use of nutrition labels according to sex and age

Stratification factors	Cases / Non-cases	Use of nutrition labels			P for trend****	P for interaction*****
		Unaware group*	Aware only group**	Aware and use group***		
At age <55 yrs						
Men	231 / 12,242	1.00 (ref)	1.80 (1.14-2.84)	1.76 (1.05-2.94)	0.07	0.03
Women	92 / 6,603	1.00 (ref)	0.87 (0.29-2.58)	0.52 (0.17-1.59)	0.03	
At age ≥ 55 yrs						
Men	942 / 9,409	1.00 (ref)	0.74 (0.61-0.89)	0.56 (0.37-0.83)	<0.001	0.93
Women	172 / 2,389	1.00 (ref)	0.90 (0.54-1.52)	0.87 (0.30-2.54)	0.69	

Abbreviation: CKD, chronic kidney disease.

$ Adjusted for the same variables included in the multivariable model of table 2.

* A group was not aware of nutrition labels.

** A group was aware of nutrition labels but didn't use it at purchasing food items.

*** A group was aware of nutrition labels and used it at purchasing food items.

**** P for trend was tested from model including the ordinal variable of nutrition labels use as a continuous term and using the Wald test of it.

***** P for interaction was tested using the cross-product term between use of nutrition labels and stratification factors.
